# Correction to: Boundary Effects Cause False Signals of Range Expansions in Population Genomic Data

**DOI:** 10.1093/molbev/msae143

**Published:** 2024-07-18

This is a correction to: Petri Kemppainen, Rhiannon Schembri, Paolo Momigliano, Boundary Effects Cause False Signals of Range Expansions in Population Genomic Data, *Molecular Biology and Evolution*, Volume 41, Issue 5, May 2024, msae091, https://doi.org/10.1093/molbev/msae091.

In the originally published version of this manuscript, the link to the data given in the Data Availability section was incorrect.

This has been corrected.

